# Supplementary material for: Adefovir Dipivoxil as a Therapeutic Candidate for Medullary Thyroid Carcinoma: Targeting RET and STAT3 Proto-Oncogenes
Source: Cancers (Basel). 2023 Apr 5;15(7):2163. doi: 10.3390/cancers15072163 (PMC10093259; doi:10.3390/cancers15072163)
Supplement: Supplementary file 1 [file cancers-15-02163-s001.zip › cancers-2300890 - supplementary.pdf]

Supplementary Materials: Adefovir Dipivoxil as a Therapeutic Candidate for Medullary Thyroid Carcinoma: Targeting RET and STAT3 Proto-Oncogenes

Tariq Alqahtani, Vishnu Kumarasamy, Sahar Saleh Alghamdi, Rasha Saad Suliman, Khalid Bin Saleh [2,7], Mohammed A. Alrashed, Mohammed Aldhaeefi and Daekyu Sun

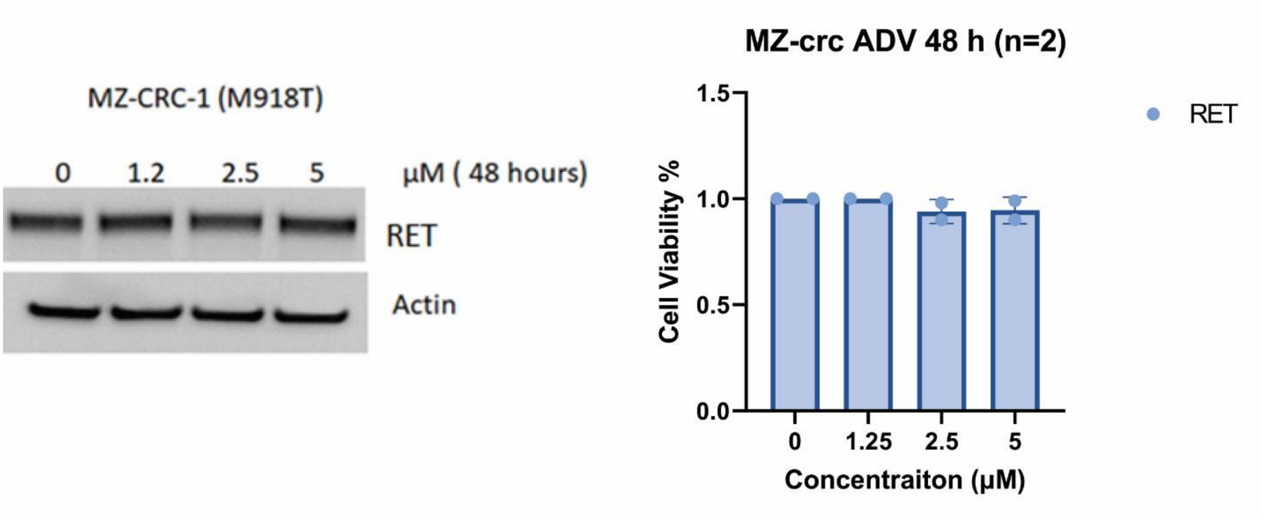

Figure S1. The effect of adefovir dipivoxil on MZ-Crc-1 (M918T) after 48 h treatment withvarious concentration.

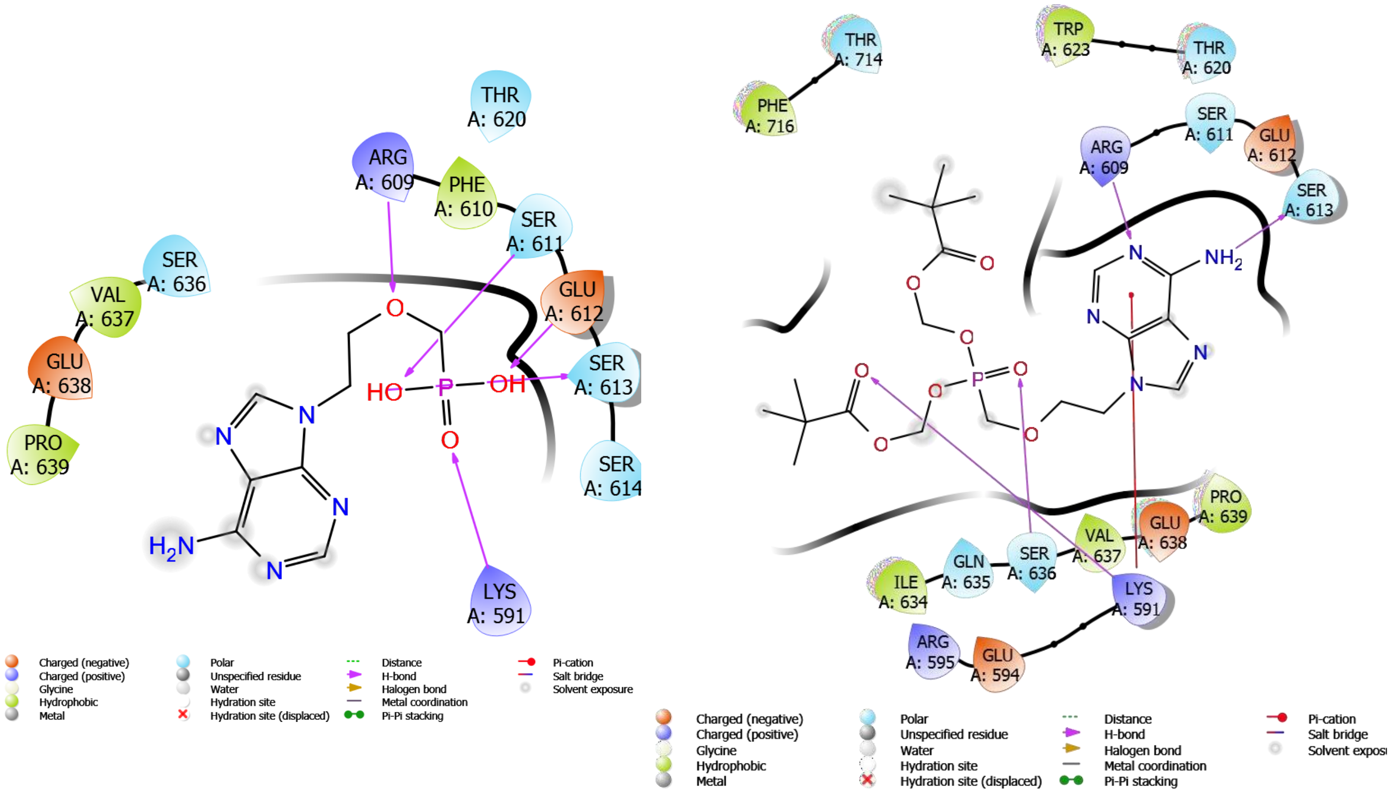

Figure S2. The 2D interactions of Adefovir and Adefovir dipivoxil at STAT3 SH2 bindingpocket.

Uncropped Western Blots (Figure S3)

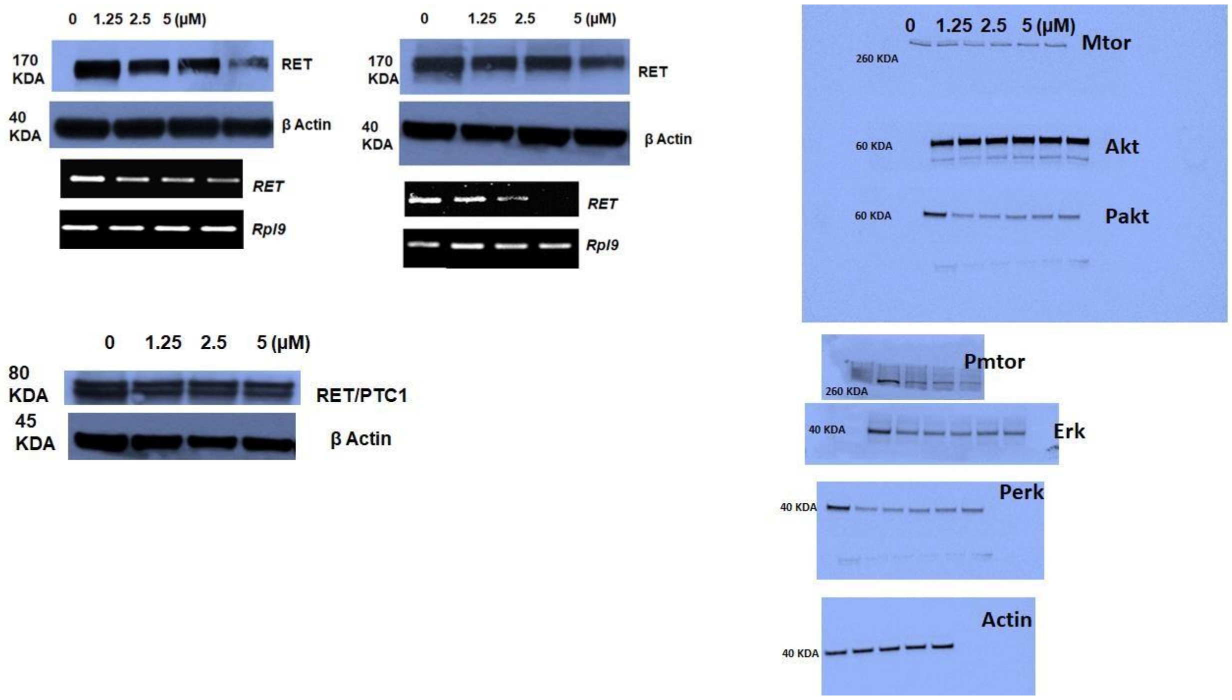

Uncropped Western Blots (Figure S4)

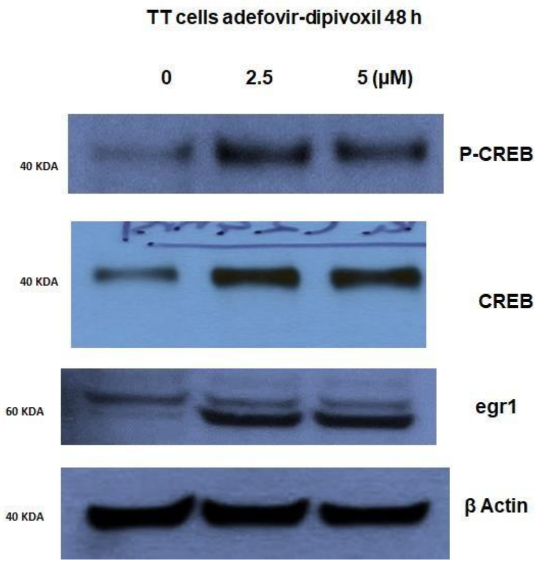

Uncropped Western Blots (Figure S5)

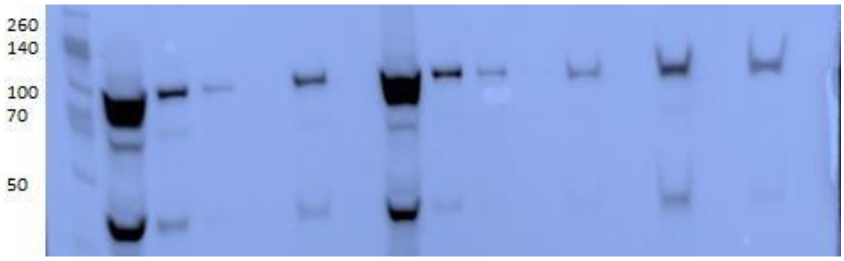

Uncropped Western Blots (Figure S6)

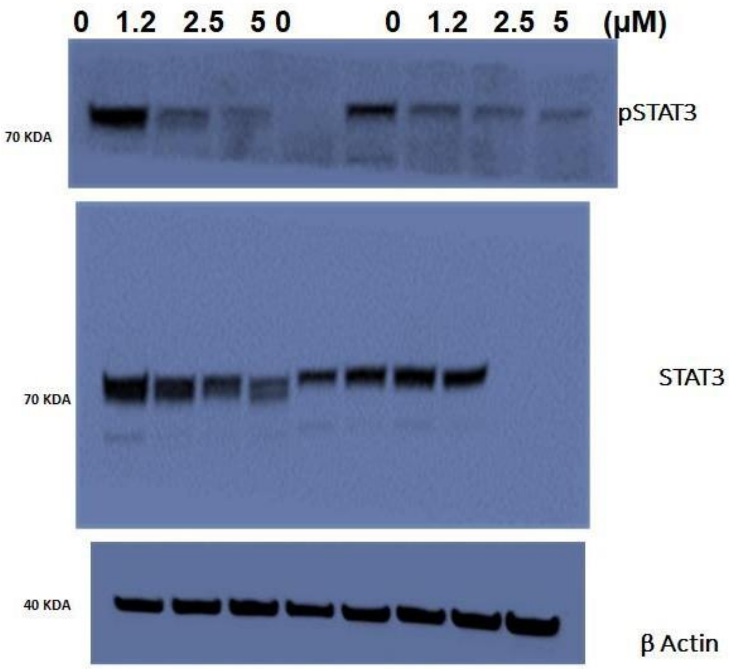

Uncropped Western Blots (Figure S7)

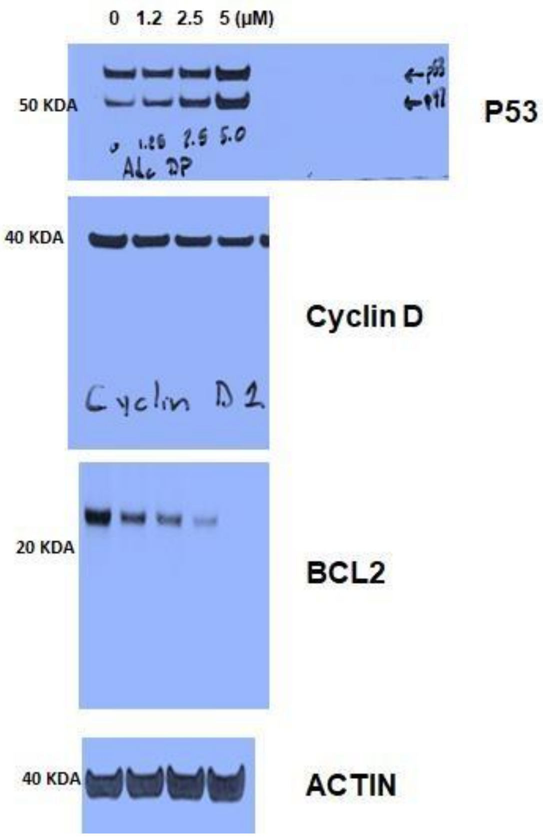

Uncropped Western Blots (Figure S8A)

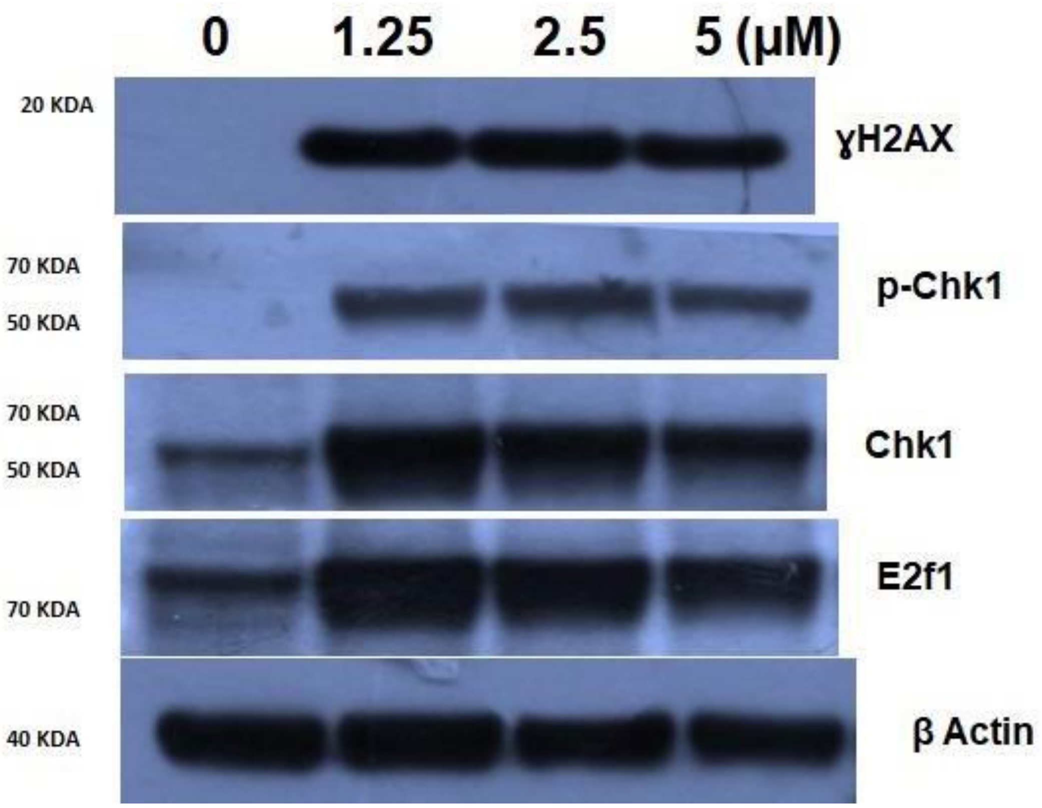

Uncropped Western Blots (Figure S8B)

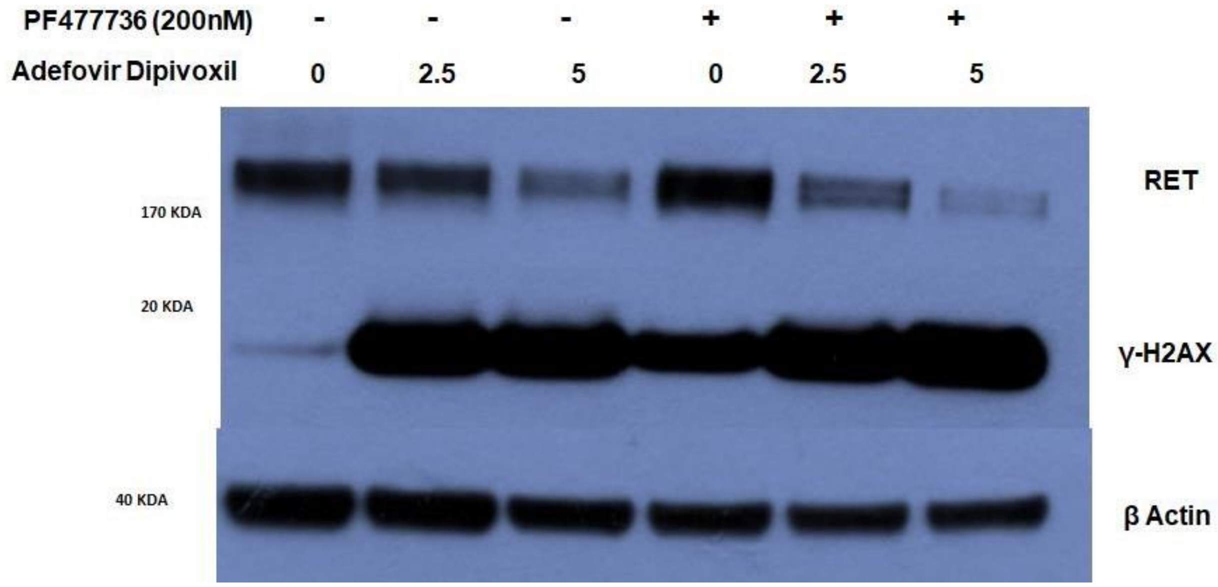

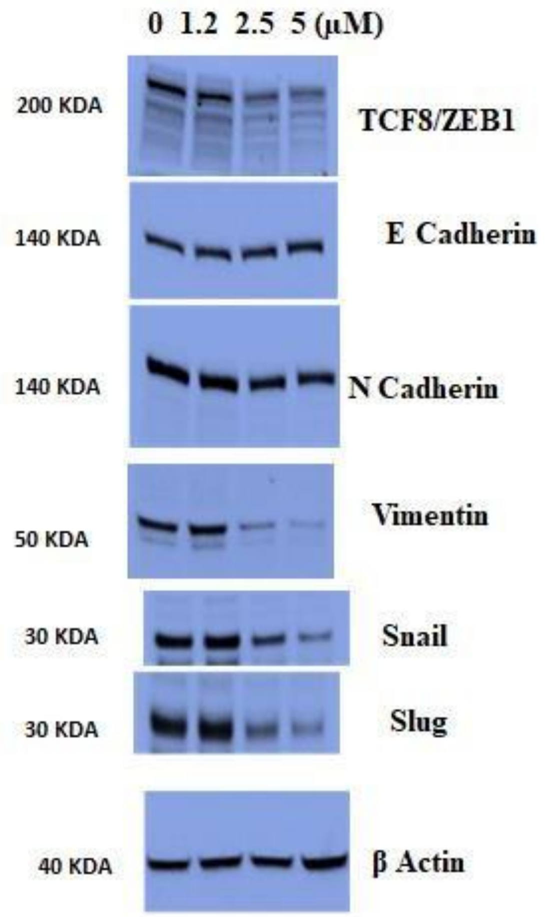

Uncropped Western Blots (Figure S9)
